# Compatibility of insecticides and fungicides with the zoophytophagous mirid predator *Nesidiocoris tenuis*

**DOI:** 10.1371/journal.pone.0187439

**Published:** 2017-11-02

**Authors:** Mohammad Ali Ziaei Madbouni, Mohammad Amin Samih, Jawwad A. Qureshi, Antonio Biondi, Peyman Namvar

**Affiliations:** 1 Department of Plant Protection, College of Agriculture, Vali-e-Asr University of Rafsanjan, Rafsanjan, Iran; 2 University of Florida/IFAS, Department of Entomology and Nematology, Indian River Research and Education Center, Fort Pierce, FL, United States of America; 3 University of Catania, Department of Agriculture, Food and Environment, Via Santa Sofia, Catania, Italy; 4 Plant Protection Research Department, South Kerman Agricultural and Natural Resources Research and Education Center, AREEO, Jiroft, Iran; Institut Sophia Agrobiotech, FRANCE

## Abstract

*Nesidiocoris tenuis* (Reuter) (Hemiptera: Miridae) is an effective predator of multiple pests of vegetable crops, such as thrips, mites, aphids, whiteflies, leafminers. It is mass-reared and released for augmentative biocontrol programs mainly aimed at controlling whiteflies and *Tuta absoluta* (Meyrick) (Lepidoptera: Gelechiidae) in greenhouses and open field. We evaluated the lethal and sublethal toxicity upon *N*. *tenuis* adults of label doses of three insecticides (pyriproxyfen, spirotetramat, cypermethrin) and seven fungicides (benomyl, chlorothalonil, copper oxychloride, cyazofamid, fluopicolide + propamocarb hydrochloride (FPH), penconazol, trifloxystrobin) commonly used in various crops. Two exposure routes were tested: (i) contact with dry residues of insecticides or fungicides on tomato sprouts and (ii) multiple exposure to these chemicals via topical sprays on adults which foraged on treated sprouts; and fed on treated eggs of *Ephestia kuehniella* (Zeller) (Lepidoptera: Pyralidae) simultaneously. Mortality and reproductive capacity were investigated as indicators of lethal and sublethal effects on *N*. *tenius*. The tested insecticides and fungicides were all classified as harmless when predator was exposed only to the dry residues of each. However, the multiple exposure to either cypermethrin, benomyl, chlorothalonil, copper oxychloride or trifloxystrobin caused significant mortality of *N*. *tenuis* adults. Cypermethrin also significantly reduced its reproductive capacity. Interestingly, *N*. *tenuis* produced a higher number of progeny when exposed to fungicides penconazol and FPH in both exposure scenarios. Overall, findings suggest that the two insecticides, pyriproxyfen and spirotetramat but not cypermethrin, and all tested fungicides can be considered compatible with *N*. *tenuis*.

## Introduction

Integrated Pest Management (IPM) strategies, employing both biological control agents and pesticides are preferred compared to pesticide only approach for sustainable production systems [1,2]. The inclusion of broad spectrum chemicals in pest management, which also rely on biological control, can negatively impact the role of natural enemies [3–5]. Therefore, the cognition of side effects of pesticides on natural enemies is critical to increase effectiveness of natural enemies and assess the suitability of pesticides for IPM [1]. This is gained through the evaluation of lethal and sublethal effects of pesticides on natural enemies. Lethal effects are acute toxicity or direct effects of pesticides that cause mortality, whereas sublethal effects could impair the physiology (e.g. neurophysiology, development, longevity, fecundity and sex-ratio) and the behavior (e.g. mobility, orientation, feeding, host searching, oviposition and mating) of natural enemies [1,6]. In addition to pests, disease control is another important consideration for profitable crop production and commonly achieved through chemical control. Fungal pathogens are responsible for several important diseases, such as the necrotrophic fungi *Botrytis cinerea* and *Alternaria solani*, the oomycete *Phytophthora infestans* and the vascular wilt fungus *Fusarium oxysporum* f. sp. lycopersici [7,8]. Fungicides are often used for disease suppression during different crop stages, such as in tomatoes [3]. Assessment of the potential non-target effects of fungicides is rarely studied; however, their understanding is important for IPM and sustainable production systems.

*Nesidiocoris tenuis* (Reuter) (Hemiptera: Miridae) is an omnivorous insect that is also a common predator of multiple pests in tomato and other agricultural crops in the Mediterranean region [9–11]. It is also mass-reared and released mainly in protected crops in augmentative biocontrol programs aimed at controlling whiteflies (Hemiptera: Aleyrodidae) and, more recently, the invasive South American tomato Pinworm *Tuta absoluta* (Meyrick) (Lepidoptera: Gelechiidae) [12–17]. Beside these two pests, *N*. *tenuis* also contributes to control of thrips, mites, aphids, spider mites, leafminers, and some other lepidopteran pests in greenhouses and field [17–19].

We evaluated the toxicity of several common pesticides and fungicides used in tomato crops for the control of whiteflies and various microbial plant diseases, respectively (Table 1). Some of these chemicals are also used in fruits crops, for example citrus or deciduous fruits which are colonized by some other mirid species. Lethal and sublethal effects of these chemical on *N*. *tenuis* adults and their reproductive potential were assessed by (i) exposing adults to dry toxic residues on the tomato plant sprouts to simulate situation where predators are released after spraying, and (ii) multiple exposure: using topical sprays on adults, that then foraged on sprouts containing dry toxic residues of the chemicals and fed on treated prey, to simulate the worst case scenario in which pesticides applications are carried out once all organisms are well established in the crop.

**Table 1:** Details of insecticides and fungicides tested for their non-target effects on *Nesidiocoris tenuis*.

Active ingredient	Trade name	Maximum field rate	Chemical family	Mode of action(s)	Crops	Target organism(s)
Cypermethrin	Cypermethrin Aria^®^	17.5 ml hl^-1^	Pyrethroids	Sodium channel modulator	Cotton, field corn, sweet corn, eggplant, pepper (bell & non-bell), tomato, head lettuce, head and stem brassica, soybean, succulent, peas and beans, root and tuber crops, pecans	Bollworms, leafrollers, snout beetles, fruit flies, codling moth, cutworm, armyworm, stalk borers, stink bugs, leafhoppers, thrips
Pyriproxyfen	Admiral^®^	75 ml hl^-1^	Pyridine	Juvenile hormone mimics	Tomato, cucurbits, peppers, cotton, citrus, mango and olives	Whiteflies and scales
Spirotetramat	Movento^®^	96 ml hl^-1^	Tetronic and tetramic acid	Inhibitors of acetyl CoA	Tomato, eggplant, peppers, strawberry, lettuce, cucurbits, leafy vegetables, onions, potatoes, sweet potatoes, beans, peas, sweet corn, citrus, grapes, mangoes, passionfruit, pome fruit, stone fruit, cotton, chicory, endive and radicchio	Aphids, whiteflies and thrips
Benomyl	Benomyl Golsam^®^	50 g hl^-1^	Benzimidazoles	Inhibitor of ß-tubuline assembly in mitosis	Tomato, cucurbits, peppers, citrus, mangoes, peas, roses, sugarcane, sunflower, tobacco seedlings, grapes, wheat, gladioli, groundnuts, bananas, peaches, apricots, plums, apples and avocados	Fungal disease
Chlorothalonil	Daconil^®^	213 ml hl^-1^	Chloronitriles	Multi-site contact activity	Tomatoes, potatoes, vegetables, cereals, stone fruit, peanuts, bananas, turf, coffee, and many other crops	Fungal disease
Copper oxychloride	Copper oxychloride Aria^®^	250 g hl^-1^	Inorganic	Multi-site contact activity	Tomato, potatoes, cucurbits, lettuce, peas, onions, apricots, cherries, peaches, nectarines, plums, almonds, apricots, cherries, nectarines, peaches, apples, pears, avocados, citrus, durians, guavas, hazelnuts, litchi, macadamias, mangoes, mangosteens, passionfruit, rambutans, vines, walnuts, bananas, blackcurrants, brassicas, capsicums, carrots, celery, ornamentals, parsnips, red beet, rhubarb, silver beet, spinach, strawberries, tobacco and seed beds	Fungal and bacterial diseases
Cyazofamid	Ranman^®^	50 ml hl^-1^	Cyano-imidazole	Quinone inside Inhibitor	Tomato, potato, cucurbits vegetables and carrots	Late blight
Fluopicolide + Propamocarb hydrochloride	Infinito^®^	160 ml hl^-1^	Pyridinylmethyl-benzamides + carbamates	Delocalisation of spectrin-like proteins + cell membrane permeability	Tomato and Potatoes	Late blight
Penconazol	Penconazol Aria^®^	12.5 ml hl^-1^	Triazoles	DeMethylation Inhibitors	Tomato, apples, pears, grapes, peas and brussels sprouts	Powdery mildew and scab
Trifloxystrobin	Flint^®^	20 ml hl^-1^	Oximino-acetates	Interrupting electron transfer at Qo center of cytochrome bc1 in the mitochondria of fungal cells	Tomatoes, eggplant, peppers, cucurbits, mangoes, grapes, pome fruit and stone fruit	Fungal disease

## Materials and methods

### Experimental insects

No permit or specific permission was required, because these studies did not involve endangered or protected species. *Nesidiocoris tenuis* used to initiate colony were collected from a pesticide-free tomato field in Jiroft (Iran) in May, 2014. The colony was maintained on pesticide-free tomato plants var. Hengam (Polaris Seeds, USA) and with an *ad libitum* supply of Mediterranean flour moth, *Ephestia kuehniella* (Zeller) (Lepidoptera: Pyralidae) eggs, often used as factitious prey [17]. These eggs were collected from E. *kuehniella* colony reared in the laboratory at 25 ± 1 ^o^C, 65 ± 10% RH, and 16:8 h L:D photoperiod. Eggs were kept in the freezer until used. The potted tomato plants with *N*. *tenuis* were kept inside the mesh cages (70 cm×60cm×60cm) in growth chamber at 25 ± 1 ^o^C, 65 ± 10% RH, and 16:8 h L:D photoperiod. Adults used in the experiments were fourth generation progeny derived from field collected specimens and were 1–2 day old at the beginning of the experiments.

#### Insecticides and fungicides

Three commercial insecticides and seven fungicides commonly used in tomato and other crops were tested (Table 1). Tap water was used to prepare the treatment solutions and to spray the untreated control. All pesticides were stored and applied following their label guidelines, and maximum recommended doses were used in the solutions simulating final application volume of 1000 L ha^-1^ (3 plants per square meter).

For dry residue exposure plants were sprayed using a 0.6 liter hand held sprayer (Canyon^®^, Northern Ireland, UK) with nozzle of the sprayer directed toward the plants from a distance of 0.5 m until runoff, resulting in a complete and uniform distribution of solution on the plant surface [20]. For the multiple exposure experiment, plants were sprayed with hand held sprayer as described above, and adults of *N*. *tenius* and eggs of *E*. *kuehniella* were sprayed using a Potter Precision Spray Tower (Burkard Manufacturing Co. Ltd) ensuring a uniform distribution of the deposit (1.5–1.8 mg cm^−2^) on all the treated surfaces [21].

#### Predator exposure to insecticides and fungicides

The experiments were conducted at the Department of Plant Protection, Agricultural and Natural Resources Research Center of southern Kerman (Iran) under controlled environment, using incubators at the same environmental conditions described for the colony maintenance. To avoid overestimation of toxic effects that usually occur when inert materials are employed as substrate, we used pesticide-free tomato plants (35-day old and 30 cm high) [6] of the same variety of those used in the rearing.

Five tomato plants/replicates were sprayed for each treatment. After spray, plants were allowed to dry for 1 h and then the upper plant part (approx.15 cm, hereafter “sprout”) was cut and placed into an experimental ventilated arena made out of two superposed plastic glasses. The top glass (430 ml, length: 11 cm) was pricked on its bottom to allow tomato plant stem to reach the water present in a second (bottom) glass (200 ml, 8 cm long) [6].

For dry residual exposure bioassay, five females and five males (1–2 d old) of *N*. *tenuis* were released into each arena containing tomato sprout with dry residues. Untreated *E*. *kuehniella* eggs were provided daily as food at the rate of 50 eggs per predator and a water source was offered ad libitum in a 1.5 mL Eppendorf tube sealed with cotton.

In the multiple exposure experiment in addition to establishing the dry residues on tomato sprouts, 1–2 day old adults of *N*. *tenuis* and *E*. *kuehniella* eggs were sprayed with pesticides solutions and water (as untreated control) in the Potter tower [22,23]. For this, five females and five males of *N*. *tenuis* or 3500 *E*. *kuehniella* eggs (50 egg×10 predator×7 days) per replicate were placed on a polyethylene mesh (220 × 331μm), which permitted excess liquid run off. Predators and *E*. *kuehniella* eggs were then placed with treated sprouts in the arenas described above. Treated *E*. *kuehniella* eggs were provided daily at the rate of 50 eggs per predator and a clean water source was offered ad libitum in a 1.5 mL Eppendorf tube sealed with cotton. Pyriproxyfen and spirotetramat did not produce significant lethal and sublethal effects in the dry residue experiment. Based on the effects of cypermethrin on non-targets in other studies [24, 25], it was included as positive control in the multiple exposure experiment to ensure that effects were observed and bioassay was reliable and tested specimens exposed to the toxicants effectively.

#### Evaluation of lethal and sublethal effects

To record the number of dead predators, observations were made daily during the 7 days after the release. Predators were considered dead when they remained immobile after being touched with a fine paintbrush. Five replicates were carried out. *Nesidiocoris tenuis* females, as most mirid species, can feed on tomato sprouts and do oviposit into vegetal tissues [26]. Therefore, to assess the reproduction capacity, surviving adults in both experiments were removed from the test arena after 7 days of exposure and placed on similar tomato sprouts that have not received any application of the chemicals. The number of emerged progeny was checked every two days for the following 12 days. To avoid any potential cannibalism, nymphs were removed from of the experimental arena at each observation.

### Data analysis

Data were analyzed for normality and homogeneity of variance using Kolmogorov–Smirnov D test. Non-normal data were transformed prior to analysis if needed. One-way Analysis of Variance (ANOVA) was used to analyze the effect of the treatment on *N*. *tenuis* mortality and offspring production (P<0.05) and Fisher's Least Significant Difference test was used for statistical separation of treatments means.

Reproduction data were used to calculate and analyze (i) total offspring produced per 5 females per replicate and any early death of predator females in a given replicate (i.e. realistic to general effect of pesticides on predator populations), (ii) Predator reproductive capacity. The number of nymphs produced was corrected by early death of a predator female in order to calculate a more accurate estimate of actual sublethal effects of the tested chemicals on reproductive capacity [6].

Survival and reproduction data including both lethal and sublethal effects from the dry residue exposure only and multiple exposure experiments were used to calculate a reduction coefficient *E*_*x*_ for pesticide *x* using the formula below [6,22]:

$$E_{x}=100-\left\{1-[\left(1-\frac{E_{mx}}{100})(1-\frac{E_{fx}}{100}\right)]\right\}$$

Where *E*_*mx*_ is the mortality corrected for the control mortality [27] and *E*_*fx*_ is the corrected reproduction estimated using the following formula:

$$E_{fx}=100-\frac{F_{x}100}{F_{c}}$$

Where *F*_*x*_ and *F*_*c*_ is the mean reproductive capacity of the female for pesticide *x* treatment and control, respectively. *E*_*x*_ values were ranked following the International Organization for Biological Control (IOBC) standards for laboratory tests: 1 = harmless (< 30%), 2 = slightly harmful (30–79%), 3 = moderately harmful (80–99%), and 4 = harmful (> 99%) [28].

## Results

### Lethal effects

**Fig 1:**
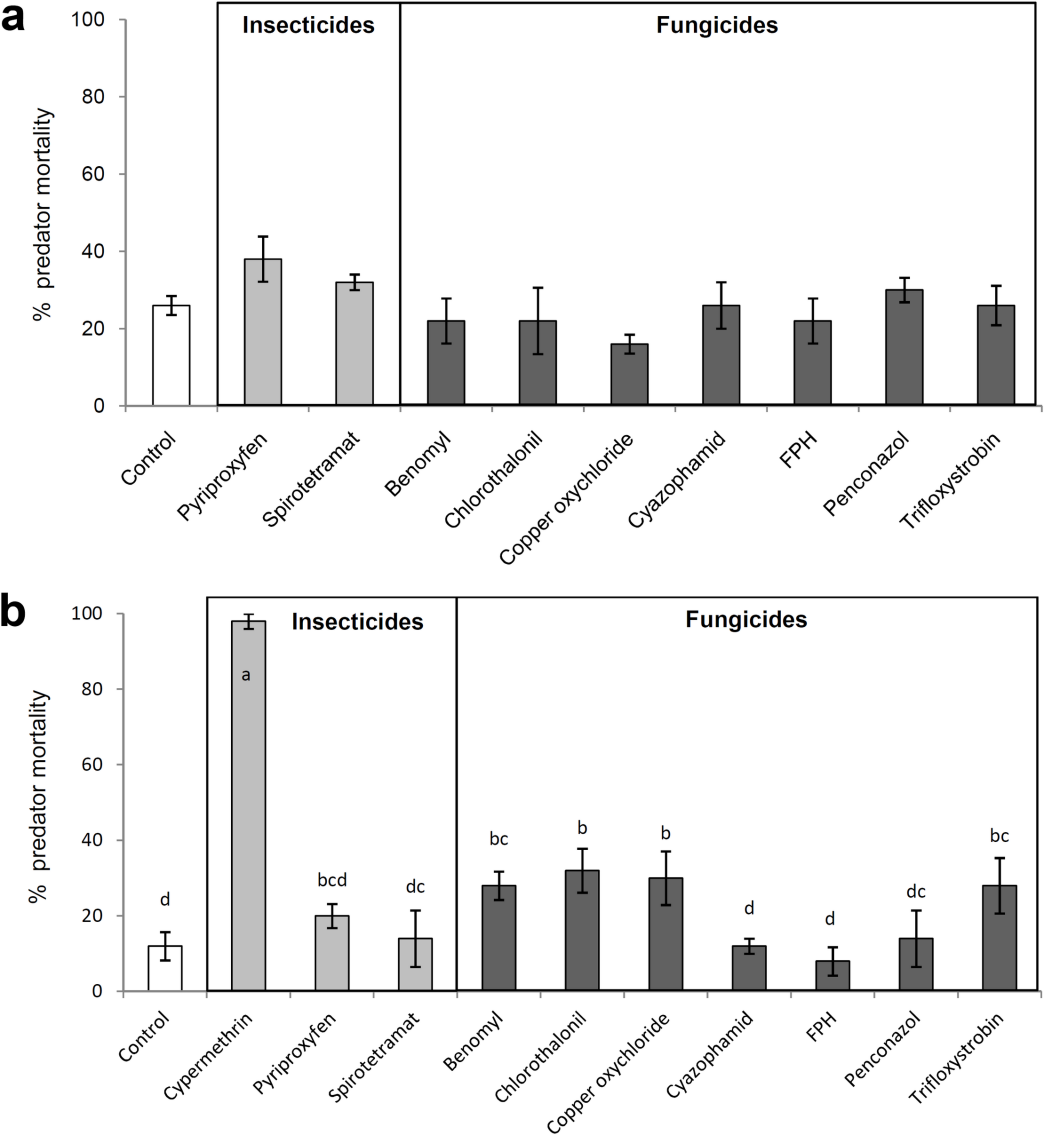
Lethal effects of insecticides and fungicides on *Nesidiocoris tenuis*. Percentage mortality (mean ± SEM) caused by insecticides and fungicides to *Nesidiocoris tenuis* after seven days of exposure of a) untreated adults to dry residues on tomato sprouts and b) treated adults to dry residues on tomato sprouts and treated eggs of the prey *Ephestia kuehniella*, simultaneously (multiple exposure). Within each subfigure, columns bearing no letters (Fig 1a) or sharing the same letter (Fig 1b) indicate no significant differences among treatments (*P* >0.05, one-way ANOVA followed by the Least Significant Difference test).

Exposure to only dry residues of any of the tested insecticides or fungicides did not cause significant mortality to adults (*F* = 1.448; df = 9, 40; *P* = 0.201, Fig 1A), although pyriproxyfen caused relatively higher mortality compared to other treatments. In contrast, there was significant treatment effect on the mortality of the adults which experienced multiple exposure to chemicals through topical application, treated plant surface and food, simultaneously (*F* = 22.266; df = 10, 44; *P*< 0.001, Fig 1B). Cypermethrin, included as positive control in the multiple exposure experiment, caused 98% mortality to *N*. *tenuis*, significantly more compared to all other treatments and control. Benomyl, chlorothalonil, copper oxychloride and trifloxystrobin also caused significant mortality of *N*. *tenuis* compared to control averaging 28, 32, 30 and 28%, respectively (Fig 1B). Mortality in other treatments insecticides or fungicides was not significantly different from mortality in the control.

### Sublethal effects

**Fig 2:**
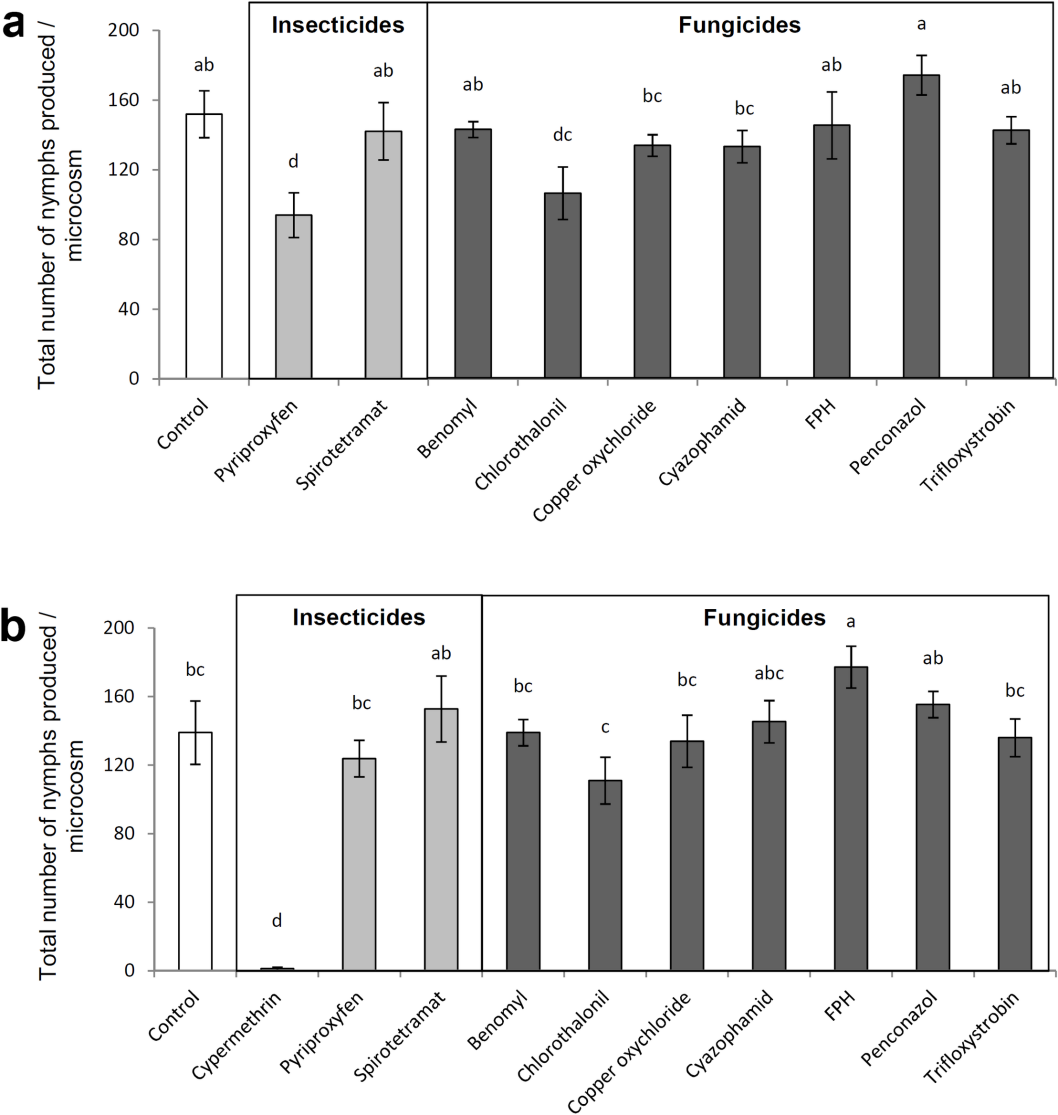
Sublethal effects of insecticides and fungicides on *Nesidiocoris tenuis* reproduction. Mean (±SEM) number of nymphs produced per five females of *Nesidiocoris tenuis* during seven days of reproduction a) females exposed to dry residues on tomato sprouts, b) females treated with topical sprays, foraged on treated sprouts and fed on treated prey *Ephestia kuehniella*, simultaneously. Within each subfigure, columns sharing a common letter indicate no significant difference between treatments (*P* >0.05, one-way ANOVA followed by the Least Significant Difference test).

Exposure to dry residues of pyriproxyfen and chlorothalonil for 7 days caused significant reduction in offspring production of females compared to control (*F* = 3.301; df = 9, 40; *P* = 0.004, Fig 2A) while all other treatments did not produce significant negative impact on reproduction. Multiple exposure to insecticides, except cypermethrin, and fungicides using treated plant, predator and prey did not reduce reproduction (*F* = 12.826; df = 10, 44; *P*< 0.001, Fig 2B).

**Fig 3:**
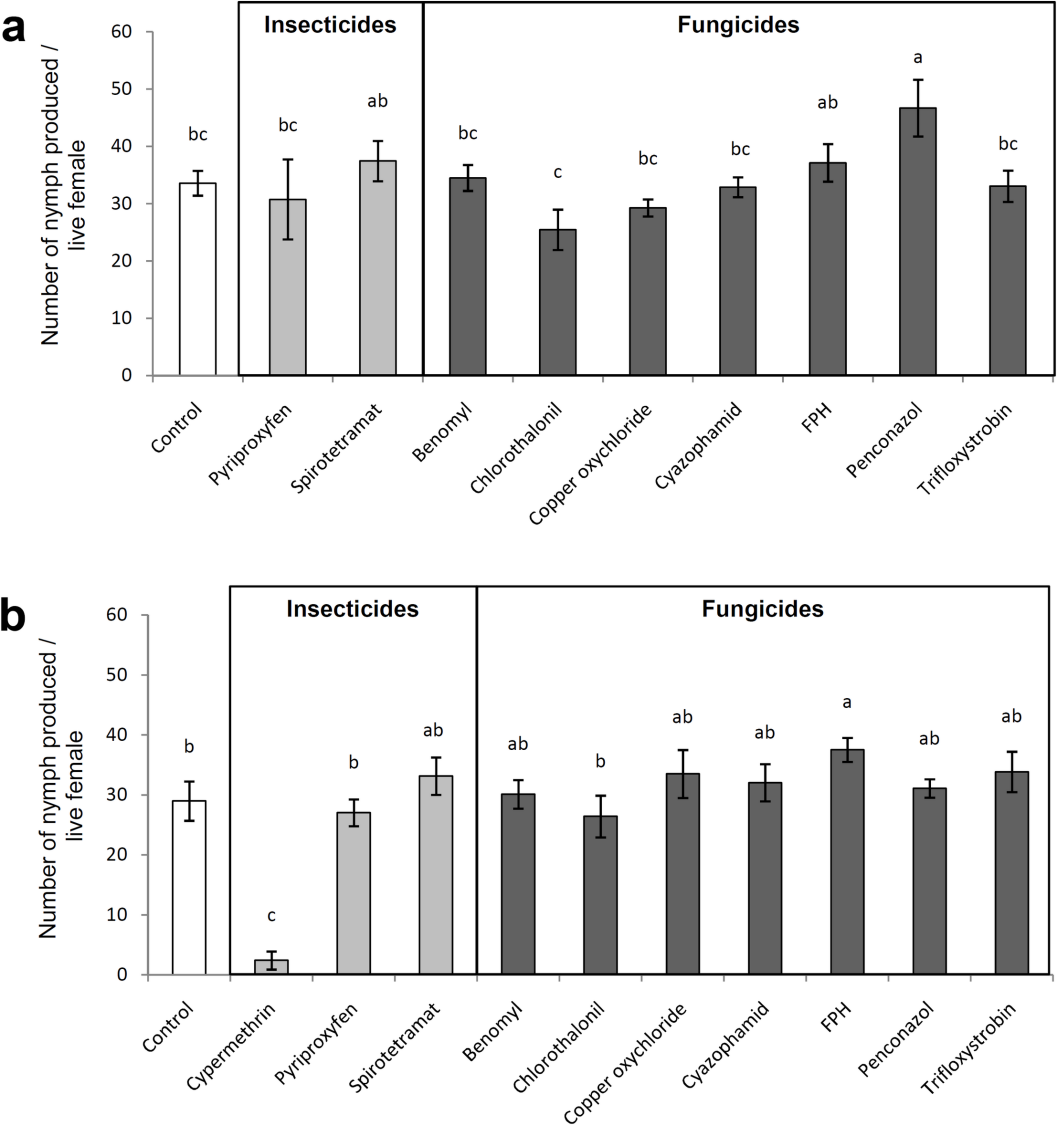
Sublethal effects of insecticides and fungicides on *Nesidiocoris tenuis* reproductive capacity. Mean (±SEM) number of nymphs produced per *Nesidiocoris tenuis* female corrected by live females during seven days of reproduction a) females were exposed to dry residues on tomato sprouts b) females were treated with topical sprays and later foraged on treated sprouts and fed on treated prey *Ephestia kuehniella*, simultaneously. Within each subfigure, columns sharing a common letter indicate no significant difference between treatments (P >0.05, one-way ANOVA followed by the Least Significant Difference test).

There was no significant negative impact of the dry residues treatment on the reproductive capacity of the female and reproduction in the treatment of penconazol was actually more compared with control (*F* = 2.43; df = 9, 40; *P* = 0.026, Fig 3A). Reproduction was reduced in the treatment of cypermethrin and increased in the treatment of fluopicolide + propamocarb hydrochloride (FPH) compared with control when females were exposed to the chemicals through multiple exposure (*F* = 10.89; df = 10, 44; P< 0.001, Fig 3B)

### Reduction coefficient (*E*_*x*_)

**Fig 4:**
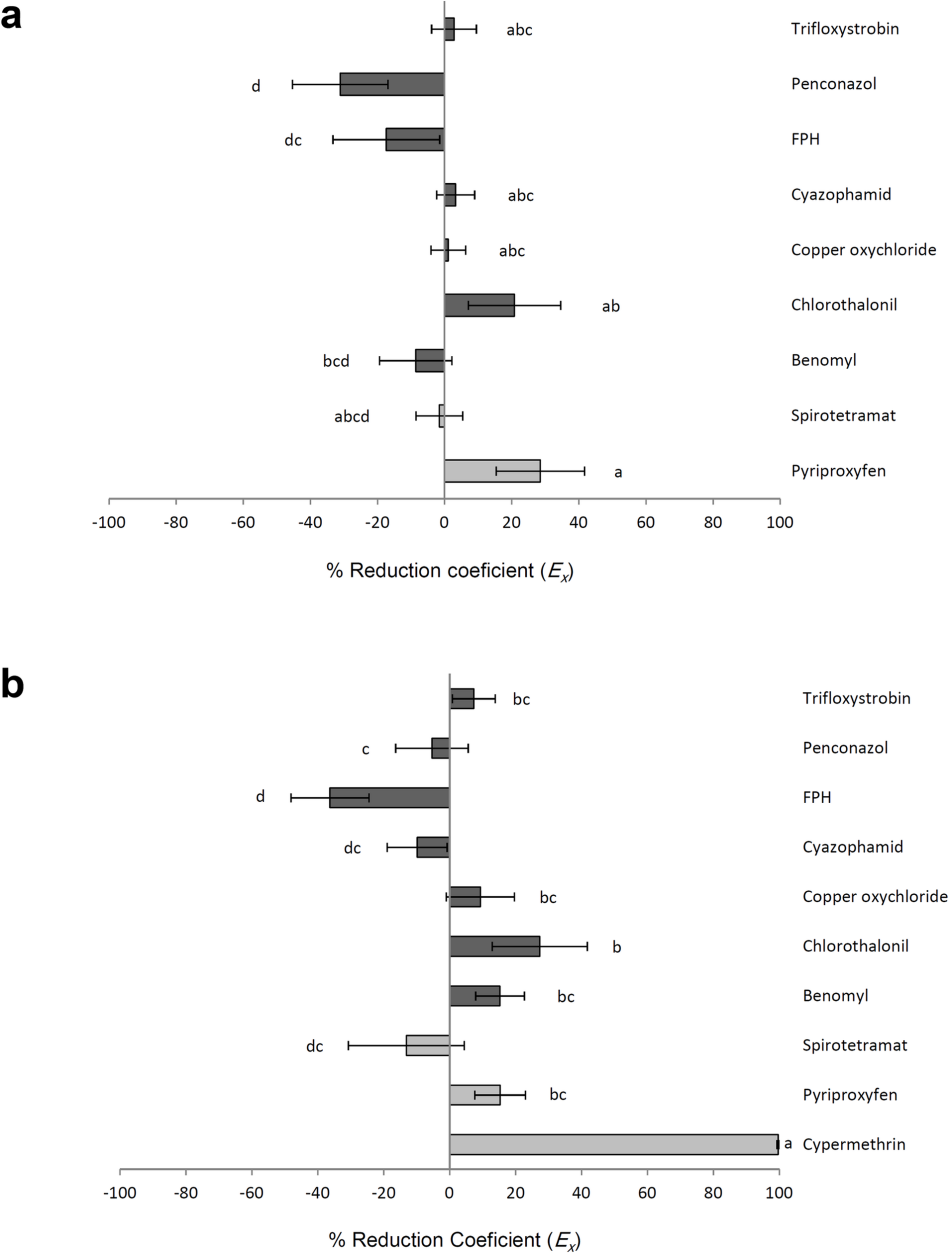
Reduction coefficient for *Nesidiocoris tenuis* exposed to insecticides and fungicides. Reduction coefficient (*E*_*x*_) of *Nesidiocoris tenuis* in insecticide and fungicide treatments integrating survival and reproduction data (a) exposed to dry residues on tomato sprouts, (b) exposed to topical sprays and later foraged on treated sprouts and fed on treated prey *Ephestia kuehniella*, simultaneously. Within each subfigure, columns sharing a common letter indicate no significant difference between treatments (P >0.05, one-way ANOVA followed by the Least Significant Difference test). Light color bars represent insecticides and dark color bars represent fungicides.

There were significant differences among treatments for dry residue experiment (F = 2.68; df = 8, 36; P = 0.018, Fig 4A) or multiple exposure experiment (F = 11.52; df = 9, 40; P< 0.001, Fig 4B). However, those showing increase in *E*_*x*_ were not significantly different in either experiment except cypermethrin which produced most effect compared with other treatments. Based on IOBC classification cypermethrin with *E*_*x*_> 90% was harmful to *N*. *tenuis* and other treatments with *E*_*x*_ lower than 30% were classified harmless (Fig 4B).

## Discussion

Exposure to dry residue of the tested insecticides did not produce significant negative effects on the predator *Nesidiocoris tenuis*. However, multiple exposures to cypermethrin through topical spray, foraging on the treated plant surfaces and ingestion of treated food caused significant mortality of adults and reduction in their reproductive capacity. Effect of this insecticide resulted in a reduction coefficient (*E*_*x*_) of > 99% which corresponds to the very harmful category by IOBC toxicity classification. Significant sublethal effects of cypermethrin on the rare survivors of *N*. *tenuis* further justified its classification as very harmful. Experiment conducted on the lacewings *Chrysoperla carnea* (Stephens) (Neuroptera: Chrysopidae) [29], *Chrysoperla externa* (Hagen) (Neuroptera: Chrysopidae) [24], *Aphidius rhopalosiph* Stefani-Pérez (Hymenoptera: Braconidae) [25] and *Phytoseiulus persimilis* Athias-Henriot (Acari: Phytoseiidae) [30] also showed harmful effects of cypermethrin on these species. Unlike natural pyrethroids, cypermethrin has a cyano group in the molecule that produce lethal effect on pests, and consequently may induce higher effects on non-target organisms [24]. Recently, the use of insect-proof nets treated pyrethroids has been proposed for the control of *T*. *absoluta* in tomato [31], thus our findings suggest a careful evaluation of their potential non-target effects on this important predator, as well as on other non-target organisms [1].

Lethal and sublethal effects of pyriproxyfen and spirotetramat on *N*. *tenuis* were minor with *E*_*x*_< 30% and therefore considered harmless. Pyriproxyfen is a juvenile hormone mimic that interferes with insect reproduction and development, and a useful insecticide against pests such as whiteflies and scales [6]. No or minor negative effects on *N*. *tenuis* makes it a useful potential product for biologically based IPM. Pyriproxyfen was also harmless to other beneficial insects such as nymphs of *Dicyphus tamaninii* Wagner (Heteroptera: Miridae) [32], adults of the seven-spotted ladybird *Coccinella septempunctata* L. (Cleoptera: Coccinellidae) [33] and adults of parasitoid *Aphytis melinus* DeBach (Hymenoptera: Aphelinidae) [34]. However, some researchers noticed mortality and reduced fertility from pyriproxyfen to parasitoids and predators. For example, Hoddle et al [35] reported significant mortality of adults and immatures of parasitoid *Eretmocerus eremicus* Rose & Zolnerowich (Hymenoptera: Aphelinidae), Biondi et al. found that *A*. *melinus* larvae exposed to this insecticide suffered strong acute toxicity and fertility reduction, and that adults tend to reject the treated hosts. Planes et al [23] reported harmful effects to *Cryptolaemus montrouzieri* Mulsant (Coleoptera: Coccinelidae). Some of these effects could be thus attributed to the tested species, their life stages, populations and experimental conditions and are important with respect to the crop system and warrant consideration. For example, effects on aphelinid parasitoids because species belonging to this family are of primary importance for the whitefly sustainable control programs [36, 37].

Spirotetramat is a systemic insecticide with potential mobility in both xylem and phloem and impacts a wide range of sucking pest complex in vegetables and fruits [38, 39]. It could also negatively impact the predators or parasitoids feeding on already intoxicated sap-sucking prey. However, we did not observe its lethal or sublethal effects on *N*. *tenuis*, neither in the worse scenario when in addition to topical contact adults foraged on treated plant surface and fed on treated eggs of *E*. *kuehniella*. Similar results were observed against the predators *N*. *tenuis* [40], *Orius armatus* (Gross) (Heteroptera: Anthocoridae) [41] and *A*. *melinus* parasitoid of scales [34, 42]. Francesena et al. [43] observed good control of the nymphs of the sweetpotato whitefly *Bemisia tabaci* (Gennadius) (Hemiptera: Aleyrodidae) with spirotetramat but low acute toxicity to the nymphal parasitoid *Eretmocerus mundus* Mercet (Hymenoptera: Aphelinidae).

All fungicides resulted in *E*_*x*_< 30% and were classified harmless although some mortality to *N*. *tenuis* was observed from benomyl, chlorothalonil, copper oxychloride and trifoxystrobin in the multiple exposure scenario. Most other studies also observed no or minor effects of these fungicides against other beneficial insects useful for biological control and IPM. For example, Yardim et al [44] reported that fungicides such as benomyl and chlorothalonil had little effect on predators. Carvalho et al. [45,46] found that benomyl was harmless to predator *Orius insidiosus* (Say) (Hemiptera: Anthocoridae) and parasitoid *Trichogramma pretiosum* Riley (Hymenoptera: Trichogrammatidae) both important biological control agents available naturally and commercially to control several pests in multiple crops including *T*. *absoluta* [47–49]. However, Michaud et al. [50] observed significant mortality to *Cycloneda sanguinea* L. (Coleoptera: Coccinellidae) larvae from benomyl, but not to *Harmonia axyridis* Pallas (Coleoptera: Coccinellidae) larvae. Larvae and nymphs of most insects end up experiencing more contact with plant surfaces and intoxicated host beside topical contact compared with adults who are able to fly and avoid contact by moving to untreated environment. Pratissoli et al. [51] reported effects of chlorothalonil on parasitism, emergence and sex ratio of *Trichogramma atopovirilia* Oatman & Platner (Hymenoptera: Trichogrammatidae) in cucurbitaceous crop. However, it was harmless to *Euseius victoriensis* (Womersley) (Acari: Phytoseiidae) [52], *T*. *pretiosum* [46] and *O*. *insidiosus* [45].

Copper oxychloride is a commonly used fungicide in vegetables such as tomato and fruits such as citrus. Our finding of its minor effects on *N*. *tenuis* was similar to other studies where it was reported safe such as to *Tamarixia radiata* (Waterston) (Hymenoptera: Eulophidae) the parasitoid of the nymphs of Asian citrus psyllid *Diaphorina citri* Kuwayama (Hemiptera: Psyllidae) 52], the predatory mite *E*. *victoriensis* [53] and the predatory lacewing *C*. *carnea* [54] which target multiple pests. However, Michaud [50] reported significant mortality of the larvae of *C*. *sanguinea* in the residual and topical bioassays with copper applied in combination with oil. Some of these effects could be associated with petroleum oil. Martinou et al [4] reported approx. 60% mortality to nymphs of predator *Macrolophus pygmaeus* (Rambur) (Hemiptera: Miridae) from copper hydroxide. Cyazofamid, FPH, penconazol and trifloxystrobin were harmless to *N*. *tenuis*. Bernard et al. [52] observed no difference in the mortality and fecundity of the predatory mite *E*. *victoriensis* from penconazole or trifloxystrobin. There is not much information on the side effects of cyazophamid and FPH on natural enemies. However, Carvalho et al., [45] reported that the lack of effects of fungicides on natural enemies may be due to the lack of the susceptible action sites in these insects. Interestingly, single and multiple exposures to Penconazol and the mixuture of Fluopicolide and Propamocarb hydrochloride had a stimulatory effect on the predator, i.e., increasing its survival and reproduction, with negative reduction coefficients. This could be due to two main reasons that should eventually be tested specifically. The first one is represented by a potential hormetic response of the predators, i.e., a chemical that is toxic at high doses can be benign at very low ones [55], and in this case fungicide label doses could have been very low as insecticides. Hormetic response of mirids to insecticides has been recently described [56]. The other reason, could be the potential curative properties that fungicides may have in the experimental conditions against entomopathogenic fungi potentially attacking both adults and developing eggs. Although entomopathogenic fungi have not been properly studied in Heteroptera predator yet, increased fertility has been registered for the flower bug, *Orius laevigatus* (Fieber) (Heteroptera: Antocoridae), and for *N*. *tenuis* following exposure to sulfur [3,6].

Mirids are an important predatory group in several crops. *Nesidiocoris tenuis* is present in most warm regions of the Northern hemisphere. There have been recent reports of its crucial role in contrasting the population of the *T*. *absoluta* [12–14]. Chemical use is also common in the systems where this pest and predator are present. Therefore, compatibility of insecticides and fungicides to *N*. *tenuis* is critical to its survival, establishment and impact on target pests. Our findings show that except cypermethrin none of the tested insecticides and fungicides were significantly harmful to *N*. *tenuis* under controlled conditions and even in worse-case scenario where predator experienced topical contact along with treated surfaces and food. Deposition pattern created using spray tower in these experiments were not very realistic of field situation where dispersion is uneven and residues expected to deteriorate by weather conditions such as temperature and rainfall. Under field conditions toxic effects of these chemicals on *N*. *tenuis* may further decrease overtime compared with those observed in these studies. Which may be useful for conservation and augmentation biological control and IPM.

## Supporting information

S1 Dataset Lethal and sublethal effects raw data. (XLSX)

## References

[1] Desneux N, Decourtye A, Delpuech JM. The sublethal effects of pesticides on beneficial arthropods. Annu Rev Entomol. 2007; 52: 81–106. doi: 10.1146/annurev.ento.52.110405.091440 16842032 10.1146/annurev.ento.52.110405.091440

[2] Roditakis E, Fytrou N, Staurakaki M, Vontas J, Tsagkarakou A. Activity of flonicamid on the sweet potato whitely *Bemisia tabaci* (Homoptera: Aleyrodidae) and its natural enemies. Pest Manag Sci. 2014; 70(10): 1460–1467. doi: 10.1002/ps.3723 24408346 10.1002/ps.3723

[3] Zappalà L, Siscaro G, Biondi A, Mollà O, Gonzalez-Cabrera J, Urbaneja A. Efficacy of sulphur on *Tuta absoluta* and its side effects on the predator *Nesidiocoris tenuis*. J Appl Entomol. 2012; 136(6): 401–409. doi: 10.1111/j.1439-0418.2011.01662.x

[4] Martinou AF, Seraphides N, Stavrinides MC. Lethal and behavioral effects of pesticides on the insect predator *Macrolophus pygmaeus*. Chemosphere. 2014; 96: 167–173. doi: 10.1016/j.chemosphere.2013.10.024 24200046 10.1016/j.chemosphere.2013.10.024

[5] Mills NJ, Beers EH, Shearer PW, Unruh TR, Amarasekare KG. Comparative analysis of pesticide effects on natural enemies in western orchards: A synthesis of laboratory bioassay data. Biol Control. 2015; 102: 17–25. doi: 10.1016/j.biocontrol.2015.05.006

[6] Biondi A, Desneux N, Siscaro G, Zappalà L. Using organic-certified rather than synthetic pesticides may not be safer for biological control agents: Selectivity and side effects of 14 pesticides on the predator *Orius laevigatus*. Chemosphere. 2012; 87(7): 803–812. doi: 10.1016/j.chemosphere.2011.12.082 22342338 10.1016/j.chemosphere.2011.12.082

[7] Arie T, Takahashi H, Kodama M, Teraoka T. Tomato as a model plant for plant-pathogen interactions. Plant Biotechnology. 2007; 24(1): 135–147.

[8] Vos CM, Yang Y, De Coninck B, Cammue BPA. Fungal (-like) biocontrol organisms in tomato disease control. Biol Control. 2014; 74: 65–81. doi: 10.1016/j.biocontrol.2014.04.004

[9] Sanchez JA, Lacasa A, Arno J, Castane C, Alomar O. Life history parameters for *Nesidiocoris tenuis* (Reuter) (Het., Miridae) under different temperature regimes. J Appl Entomol. 2009; 133(2): 125–132. doi: 10.1111/j.1439-0418.2008.01342.x

[10] Calvo FJ, Lorente M, Stansly PA, Belda JE. Release rate for a pre-plant application of *Nesidiocoris tenuis* for *Bemisia tabaci* control in tomato. BioControl. 2012; 57(6): 809–817. doi: 10.1007/s10526-012-9455-1

[11] Perdikis D, Arvaniti K. Nymphal development on plant vs. leaf with and without prey for two omnivorous predators: *Nesidiocoris tenuis* (Reuter, 1895) (Hemiptera: Miridae) and *Dicyphus errans* (Wolff, 1804) (Hemiptera: Miridae). Entomol Gen. 2016; 35(4): 297–306. doi: 10.1127/entomologia/2016/0219

[12] Campos MR, Biondi A, Adiga A, Guedes RNC, Desneux N. From the Western Palaearctic region to beyond: *Tuta absoluta* 10 years after invading Europe. J Pest Sci. 2017; doi: 10.1007/s10340-017-0867-7

[13] Zappalà L, Biondi A, Alma A, Al-Jboory IJ, Arnò J, Bayram A, et al Natural enemies of the South American moth, *Tuta absoluta*, in Europe, North Africa and Middle East, and their potential use in pest control strategies. J Pest Sci. 2013; 86: 635–647. doi: 10.1007/s10340-013-0531-9

[14] Biondi A, Guedes RNC, Wan FH, Desneux N. Ecology, Worldwide Spread and Management of the Invasive South American Tomato Pinworm, *Tuta absoluta*: Past, Present, and Future. Annu Rev Entomol. 2018; doi: 10.1146/annurev-ento-031616-034933 28977774 10.1146/annurev-ento-031616-034933

[15] Urbaneja A, Tapia G, Stansly P. Influence of host plant and prey availability on developmental time and survivorship of *Nesidiocoris tenuis* (Het.: Miridae). Biocontrol Sci Technol. 2005; 15: 513–518. doi: 10.1080/09583150500088777

[16] Alomar O, Riudavets J, Castane C. *Macrolophus caliginosus* in the biological control of *Bemisia tabaci* on greenhouse melons. Biol Control. 2006; 36: 154–162. doi: 10.1016/j.biocontrol.2005.08.010

[17] Mollá O, Biondi A, Alonso-Valiente M, Urbaneja A. A comparative life history study of two mirid bugs preying on *Tuta absoluta* and *Ephestia kuehniella* eggs on tomato crops: implications for biological control. BioControl. 2014; 59(2): 175–183. doi: 10.1007/s10526-013-9553-8

[18] Calvo J, Bolckmans K, Stansly PA, Urbaneja A. Predation by *Nesidiocoris tenuis* on *Bemisia tabaci* and injury to tomato. BioControl. 2009; 54: 237–246. doi: 10.1007/s10526-008-9164-y

[19] Urbaneja-Bernat P, Alonso-Valiente M, Tena A, Bolckmans K, Urbaneja A. Sugar as nutritional supplement for the zoophytophagous predator *Nesidiocoris tenuis*. BioControl. 2013; 58: 57–64. doi: 10.1007/s10526-012-9466-y

[20] Abbes K, Biondi A, Kurtulus A, Ricupero M, Russo A, Siscaro G, et al Combined non-target effects of insecticide and high temperatures on the parasitoid *Bracon nigricans*. PLoS One. 2015; 10(9): e0138411 doi: 10.1371/journal.pone.0138411 26382245 10.1371/journal.pone.0138411 PMC4575060

[21] Biondi A, Campolo O, Desneux N, Siscaro G, Palmeri V, Zappalà L. Life stage-dependent susceptibility of *Aphytis melinus* DeBach (Hymenoptera: Aphelinidae) to two pesticides commonly used in citrus orchards. Chemosphere. 2015; 128: 142–147. doi: 10.1016/j.chemosphere.2015.01.034 25698292 10.1016/j.chemosphere.2015.01.034

[22] Urbaneja A, Pascual-Ruiz S, Pina T, Abad-Moyano R, Vanaclocha P, Montón H, et al Efficacy of some acaricides against *Tetranychus urticae* (Acari: Tetranychidae) and their side-effects on selected natural enemies occurring in citrus orchards. Pest Manag Sci. 2008; 64: 834–842. doi: 10.1002/ps.1572 18383196 10.1002/ps.1572

[23] Planes L, Catalán J, Tena A, Porcuna JL, Jacas JA, Izquierdo J, et al Lethal and sublethal effects of spirotetramat on the mealybug destroyer, *Cryptolaemus montrouzieri*. J Pest Sci. 2013; 86(2): 321–32. doi: 10.1007/s10340-012-0440-3.

[24] Rimoldi F, Schneider MI, Ronco AE. Susceptibility of *Chrysoperla externa* eggs (Neuroptera: Chrisopidae) to conventional and biorational insecticides. Environ Entomol. 2008; 37(5): 1252–1257. doi: 10.1093/ee/37.5.1252 19036204 10.1603/0046-225x(2008)37[1252:soceen]2.0.co;2

[25] Jansen JP. Side effects of insecticides on *Aphidius rhopalosiphi* (Hym.: Aphidiidae) in laboratory. Entomophaga. 1996; 41(1): 37–43. doi: 10.1007/BF02893290

[26] Biondi A, Zappalà L, Di Mauro A, Tropea Garzia G, Russo A, Desneux N, et al Can alternative host plant and prey affect phytophagy and biological control by the zoophytophagous mirid *Nesidiocoris tenuis*? BioControl. 2016; 61:79–90. doi: 10.1007/s10526-015-9700-5

[27] Abbott WS. A method for computing the effectiveness of an insecticide. J. Econ. Entomol. 1925; 18: 265–267. doi: 10.1093/jee/18.2.265a

[28] Hassan SA. Activities of the IOBC/WPRS working group pesticides and beneficial organisms. IOBC/WPRS Bull. 1994; 17: 1–5.

[29] Sarode SV, Sonalkar VU. Ovicidal effect of some insecticides against *Chrysoperla carnea*. Pesticide Res J. 1999; 11: 97–98.

[30] Oomen PA, Romeijn G, Wiegers GL. Side-effects of 100 pesticides on the predatory mite *Phytoseiulus persimilis*, collected and evaluated according to the EPPO Guideline. EPPO Bulletin. 1991; 21: 701–712. doi: 10.1111/j.1365-2338.1991.tb01304.x

[31] Biondi A, Zappalà L, Desneux N, Aparo A, Siscaro G, Rapisarda C, et al Potential toxicity of α-cypermethrin-treated nets on *Tuta absoluta* (Lepidoptera: Gelechiidae). J Econ Entomol. 2015; 108(3): 1191–1197. doi: 10.1093/jee/tov045 26470245 10.1093/jee/tov045

[32] Castane C, Arino J, Arno J. Toxicity of some insecticides and acaricides to the predatory bug *Dicyphus tamaninii* (Het.: Miridae). Entomophaga. 1996; 41(2): 211–216. doi: 10.1007/BF02764246

[33] Bozsik A. Susceptibility of adult *Coccinella septempunctata* (Coleoptera: coccinellidae) to insecticides with different modes of action. Pest Manag Sci. 2006; 62: 651–654. doi: 10.1002/ps.1221 16649191 10.1002/ps.1221

[34] Vanaclocha P, Vidal-Quist C, Oheix S, Montón H, Planes L, Catalán J. Acute toxicity in laboratory tests of fresh and aged residues of pesticides used in citrus on the parasitoid *Aphytis melinus*. J Pest Sci. 2013; 86(2): 329–336. doi: 10.1007/s10340-012-0448-8

[35] Hoddle MS, Van Driesche RG, Lyon SM, Sanderson JP. Compatibility of insect growth regulators with *Eretmocerus eremicus* (Hymenoptera: Aphelinidae) for whitefly (Homoptera: Aleyrodidae) control on Poinsettias. Biol Control. 2001; 20: 122–131. doi: 10.1006/bcon.2000.0885

[36] Drobnjaković T, Marčić D, Prijović M, Perić P, Milenković S, Bošković J. Life history traits and population growth of *Encarsia formosa* Gahan (Hymenoptera: Aphelinidae) local population from Serbia. Entomol Gen. 2016; 35: 281–295. doi: 10.1127/entomologia/2016/0183

[37] Velasco-Hernández MC, Ramirez-Romero R, Sanchez-Hernandez C. Foraging behavior of the parasitoid *Eretmocerus eremicus* under intraguild predation risk by *Macrolophus pygmaeus*. Pest Manag. Sci. 2015; 71:1346–1353. doi: 10.1002/ps.3938 25377901 10.1002/ps.3938

[38] Nauen R, Reckmann U, Thomzik J, Thielert W. Biological profile of spirotetramat (Movento®)–a new two-way systemic (ambimobile) insecticide against sucking pest species. Bayer CropSci J. 2008; 61(2): 245–277.

[39] Qureshi JA, Kostyk B, Stansly PA. Insecticidal suppression of Asian citrus psyllid *Diaphorina citri* (Hemiptera: Liviidae) vector of huanglongbing pathogens. PLoS ONE 9(12): e112331 doi: 10.1371/journal.pone.0112331 25437858 10.1371/journal.pone.0112331 PMC4249845

[40] Wanumen AC, Sanchez-Ramos I, Vinuela E, Medina P, Adan A. Impact of feeding on contaminated prey on the life parameters of *Nesidiocoris tenuis* (Hemiptera: Miridae) adults. J Insect Sci. 2016; 16: 1–7. doi: 10.1093/jisesa/iew084 27694345 10.1093/jisesa/iew084 PMC5043474

[41] Broughton S, Harrison J, Rahman T. Effect of new and old pesticides on *Orius armatus* (Gross)—an Australianpredator of western flower thrips, *Frankliniella occidentalis* (Pergande). Pest Manag Sci. 2014; 70(3): 389–97. doi: 10.1002/ps.3565 23616278 10.1002/ps.3565

[42] Garcerá C, Ouyang Y, Scott SJ, Moltó E, Grafton-Cardwell EE. Effects of spirotetramat on *Aonidiella aurantii* (Homoptera: Diaspididae) and its parasitoid, *Aphytis melinus* (Hymenoptera: Aphelinidae). J Econ Entomol. 2013; 106(5): 2126–2134. doi: 10.1603/EC12510 24224255 10.1603/ec12510

[43] Francesena N, Haramboure M, Smagghe G, Stadler T, Schneider MI. Preliminary studies of effectiveness and selectivity of Movento on *Bemisia tabaci* and its parasitoid *Eretmocerus mundus*. Commun Agric Appl Biol Sci. 2012; 77(4): 727–733. 23885443

[44] Yardim EN, Edwards CA. The infuence of chemical management of pests, diseases and weeds on pest and predatory arthropods associated with tomatoes. Agr Ecosyst Environ. 1998; 70: 31–48. doi: 10.1016/S0167-8809(97)00160-6

[45] Carvalho GA, Bueno VHP, Moura AP, Rocha LCD, Torres FZV. Side effect of pesticides on *Orius insidiosus* (Hemiptera: Anthocoridae). IOBC/WPRS Bull. 2006; 29(4): 349–353.

[46] Carvalho GA, Moura AP, Bueno VHP. Side effect of pesticides on *Trichogramma pretiosum* (Hymenoptera: Trichogrammatidae). IOBC/WPRS Bull. 2006; 29(4): 355–359.

[47] Salehi Z, Yarahmadi F, Rasekh A, Sohani NZ. Functional responses of *Orius albidipennis* Reuter (Hemiptera, Anthocoridae) to *Tuta absoluta* Meyrick (Lepidoptera, Gelechiidae) on two tomato cultivars with different leaf morphological characteristics. Entomol Gen. 2016; 36: 127–136. doi: 10.1127/entomologia/2016/0339

[48] Chailleux A, Biondi A, Han P, Desneux N. Suitability of the pest-plant system *Tuta absoluta* (Lepidoptera: Gelechiidae)-tomato for *Trichogramma* (Hymenoptera: Trichogrammatidae) parasitoids and insights for biological control. J Econ Entomol. 2013; 106: 2310–2321. doi: 10.1603/EC13092 24498728 10.1603/ec13092

[49] El-Arnaouty SA, Pizzol J, Galal HH, Kortam MN, Afifi AI, Beyssat V, Desneux N, Biondi A, Heikal IH. Assessment of two Trichogramma species for the control of *Tuta absoluta* in north african tomato greenhouses.African Entomol. 2014; 22: 801–814. doi: 10.4001/003.022.0410

[50] Michaud JP. Responses of two ladybeetles to eight fungicides used in Florida citrus: implications for biological control. J Insect Sci. 2001; 1(1);1–6. doi: 10.1093/jis/1.1.6 15455066 PMC355890

[51] Pratissoli D, Milanez AM, Barbosa WF, Celestino FN, Andrade GS, Polanczyk RA. Side effects of fungicides used in cucurbitaceous crop on *Trichogramma atopovirilia* Oatman & Platner (Hymenoptera: Trichogramatidae). Chil J Agr Res. 2010; 70(2): 323–327. doi: 10.4067/S0718-58392010000200016

[52] Hall DG. Nguyen R. Toxicity of pesticides to *Tamarixia radiata*, a parasitoid of the Asian citrus psyllid. Biocontrol. 2010; 55(5): 601–611. doi: 10.1007/s10526-010-9283-0

[53] Bernard MB, Cole P, Kobelt A, Horne PA, Altmann J, Wratten SD, et al Reducing the impact of pesticides on biological control in australian vineyards: pesticide mortality and fecundity effects on an indicator species, the predatory mite *Euseius victoriensis* (Acari: Phytoseiidae). J Econ Entomol. 2010; 103(6): 2061–2071. doi: 10.1603/EC09357 21309226 10.1603/ec09357

[54] Bengochea P, Saelices R, Amor F, Adán Á, Budia F, Estal P, et al Non-target effects of kaolin and coppers applied on olive trees for the predatory lacewing *Chrysoperla carnea*. Biocontrol Sci Technol. 2014; 24 (6): 625–640. doi: 10.1080/09583157.2014.884212

[55] Guedes RNC, Cutler GC. 2014 Insecticide-induced hormesis and arthropod pest management. Pest Manag Sci. 2014: 70(5): 690–697. doi: 10.1002/ps.3669 24155227 10.1002/ps.3669

[56] Tan Y, Biondi A, Desneux N, Gao XW. Assessment of physiological sublethal effects of imidacloprid on the mirid bug *Apolygus lucorum* (Meyer-Dür). Ecotoxicology. 2012; 21:1989–1997. doi: 10.1007/s10646-012-0933-0 22740097 10.1007/s10646-012-0933-0

